# Supplementary figures and images for: USP5 inhibits anti-RNA viral innate immunity by deconjugating K48-linked unanchored and K63-linked anchored ubiquitin on IRF3
Source: PLoS Pathog. 2025 Jan 6;21(1):e1012843. doi: 10.1371/journal.ppat.1012843 (PMC11737852; doi:10.1371/journal.ppat.1012843)

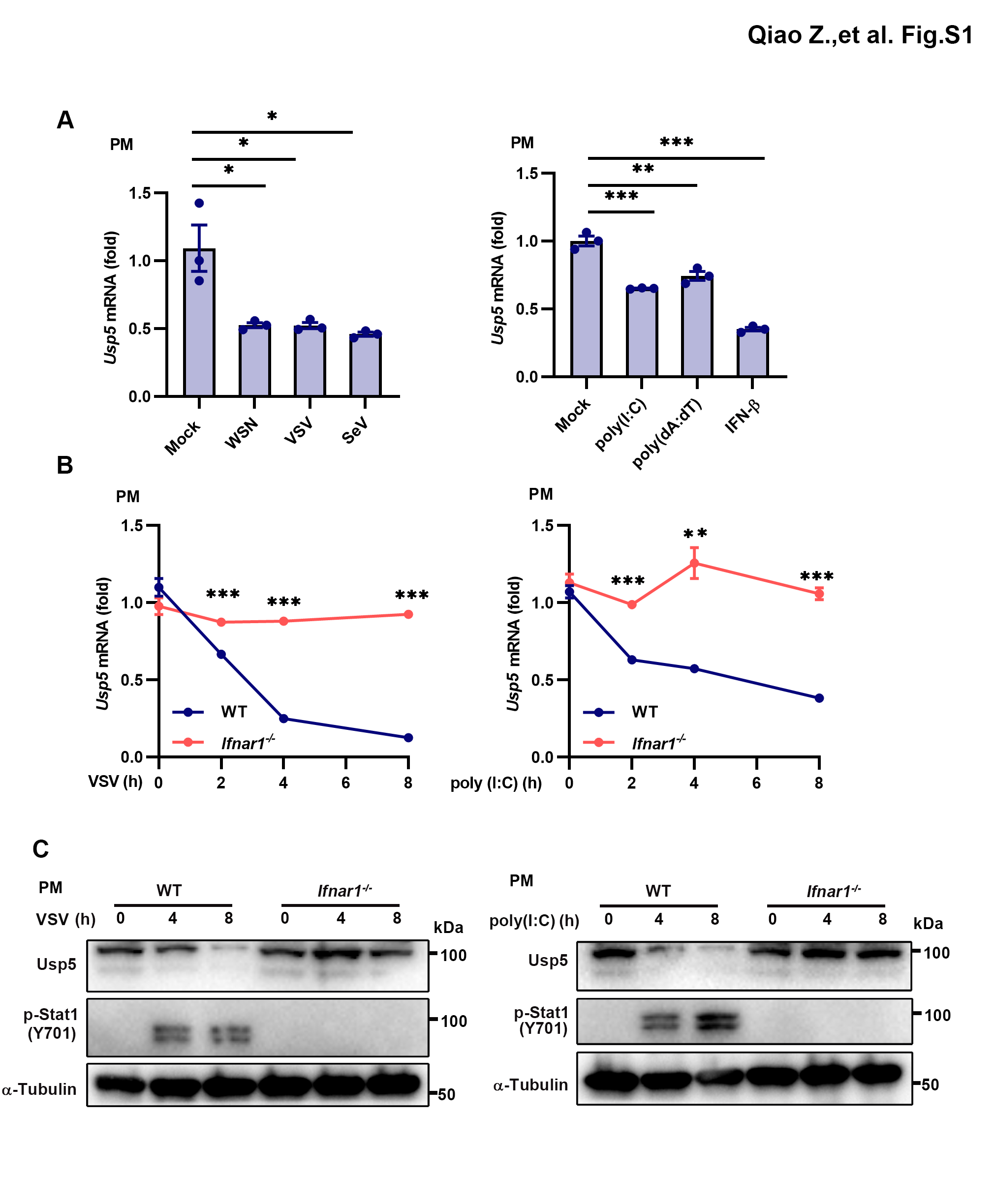

Supplement: S1 Fig — (A) RT-qPCR analysis of Usp5 mRNA expression in the PMs following infection with WSN (1 MOI), VSV (0.1 MOI), and SeV (0.1 MOI) for 12 h, transfection with poly(I:C) (1 μg/mL), poly(dA:dT) (1 μg/mL), or stimulation with IFN-β (500 U/mL) for 6 h. (B) RT-qPCR analysis of USP5 mRNA levels in the PMs from WT and Ifnar1-/- mice, following VSV infection at 0.1 MOI or poly(I:C) transfection at 1 μg/mL for 0, 2, 4, and 8 h. (C) Immunoblot analysis of Usp5 mRNA levels in PMs from WT and Ifnar1-/- mice, following VSV infection at 0.1 MOI or poly(I:C) transfection at 1 μg/mL for 0, 4, and 8 h. Data are representative of 3 independent experiments (A-C). Mean ± SEM, statistical analysis was performed using unpaired two-tailed Student’s t-test (B) or one-way ANOVA (A), *p<0.05, **p<0.01, and ***p<0.001 indicate the significant differences. (TIF) [file ppat.1012843.s001.tif]

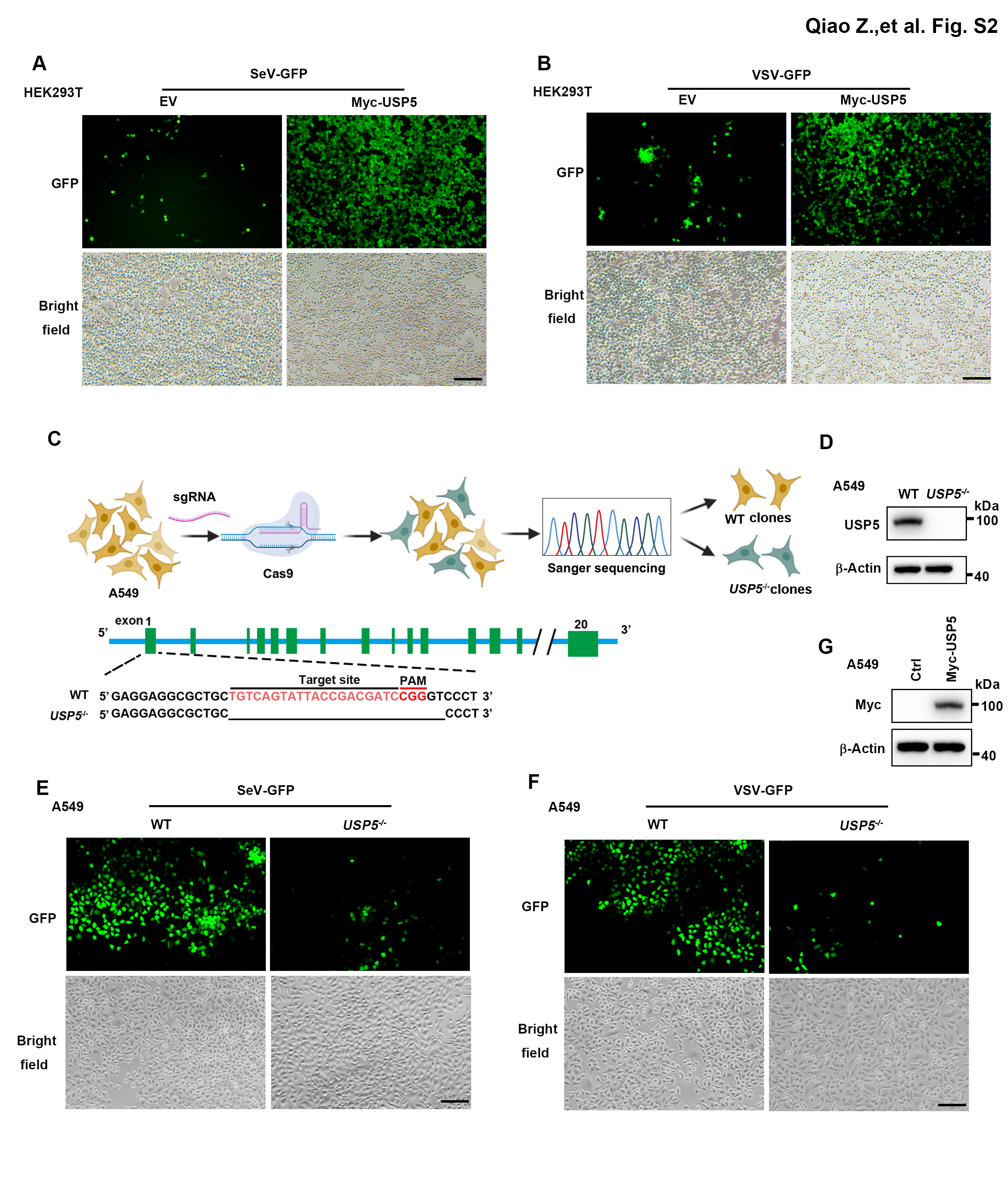

Supplement: S2 Fig — (A) Fluorescence microscopy analyses of HEK293T cells transfected with EV or Myc-USP5 for 24 h, following infection with SeV-GFP (0.1 MOI) for 8 h. Scale bar, 200 μm. (B) Fluorescence microscopy analyses of HEK293T cells transfected with EV or Myc-USP5 for 24 h, following infection with VSV-GFP (0.1 MOI) for 8 h. Scale bar, 200 μm. (C) Schematic diagram of the CRISPR/Cas9 gene editing protocol for USP5 and the Sanger sequencing results. (D) Immunoblot analysis of USP5 expression in WT and USP5-/- A549 cells. (E) Fluorescence microscopy analyses of WT and USP5-/- A549 cells infected with SeV-GFP (0.1 MOI) for 6 h. Scale bar, 200 μm. (F) Fluorescence microscopy analyses of WT and USP5-/- A549 cells infected with VSV-GFP (0.1 MOI) for 6 h. Scale bar, 200 μm. (G) Immunoblot analysis of Myc-USP5 expression in Ctrl and Myc-USP5 overexpressed A549 cells. Data are representative of 3 independent experiments (A, B, and D-G). Created with Biorender.com. (TIF) [file ppat.1012843.s002.tif]

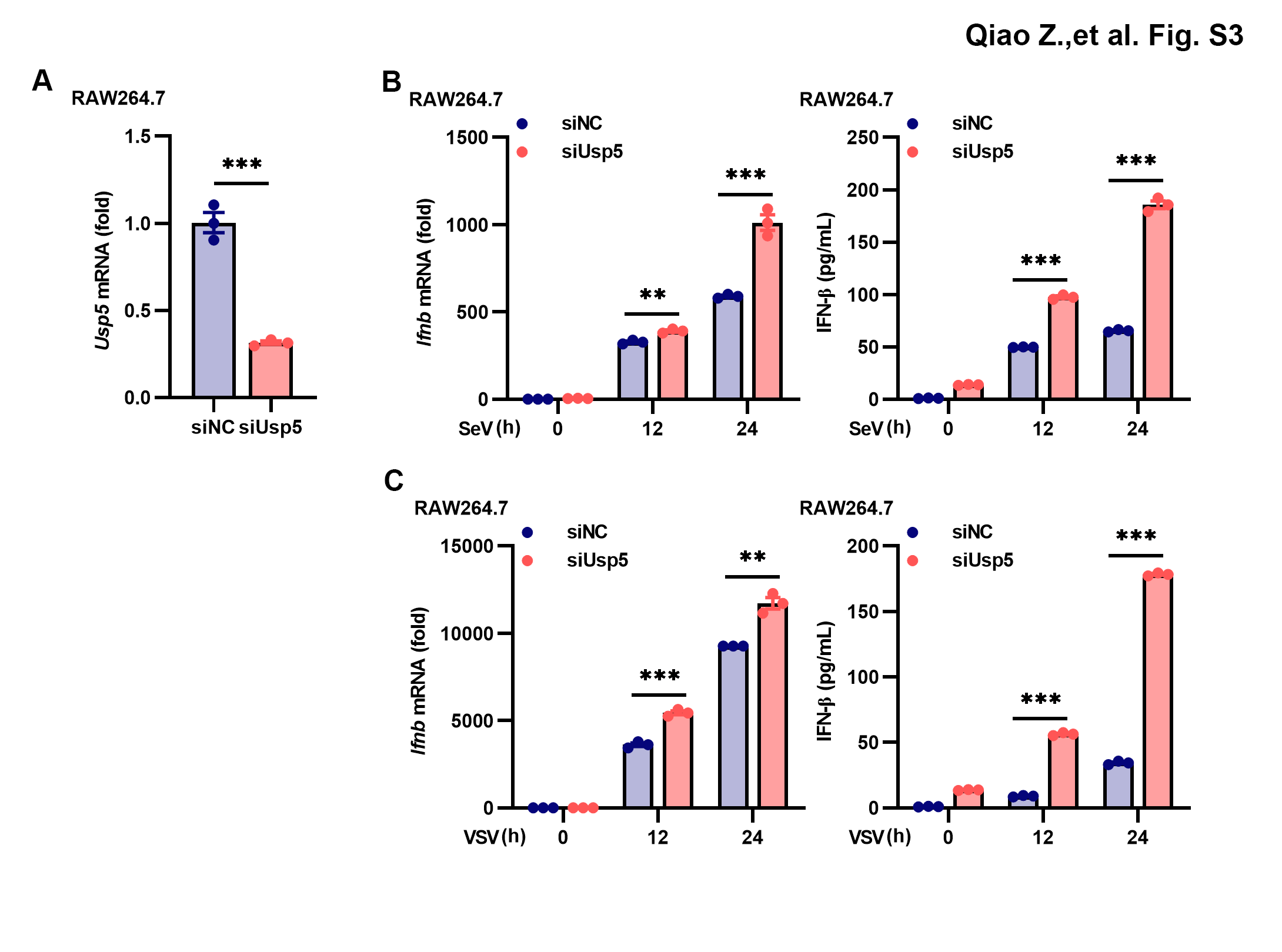

Supplement: S3 Fig — (A) RT-qPCR analysis of the RNAi efficiency targeting USP5 in RAW264.7 cells 36 h post-transfection. (B) RT-qPCR and ELISA analyses were conducted to assess Ifnb mRNA levels and IFN-β protein expression, respectively, in the control and USP5 knockdown RAW264.7 cells at 0, 12, and 24 h post-infection with SeV (0.1 MOI). (C) RT-qPCR and ELISA analyses were conducted to assess Ifnb mRNA levels and IFN-β protein expression, respectively, in control and USP5 knockdown RAW264.7 cells at 0, 12, and 24 h post-infection with VSV (0.1 MOI). Data are representative of 3 independent experiments (A-C). Mean ± SEM, statistical analysis was performed using unpaired two-tailed Student’s t-test (A-C), **p<0.01, and ***p<0.001 indicate the significant differences. (TIF) [file ppat.1012843.s003.tif]

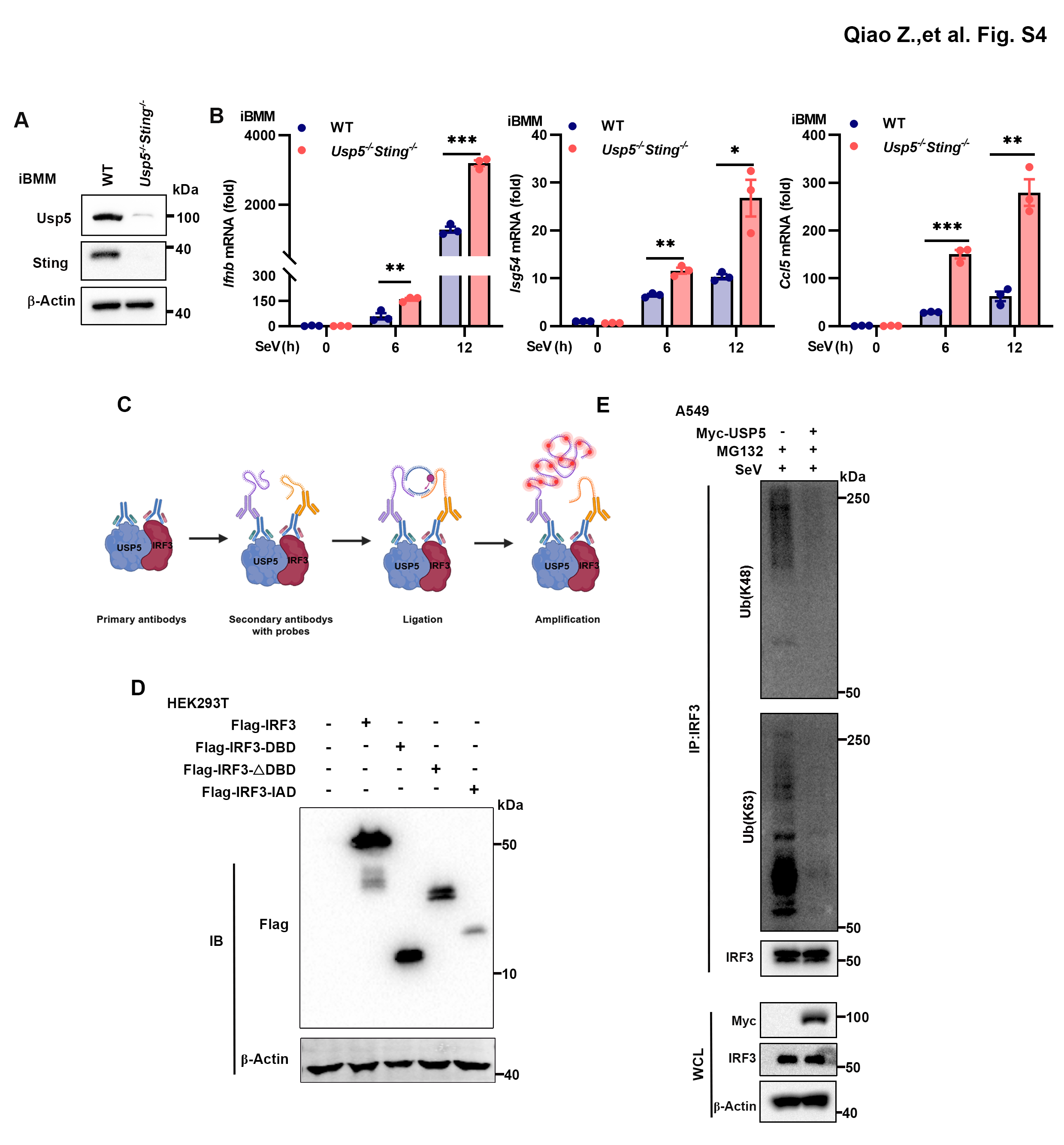

Supplement: S4 Fig — (A) Immunoblot analysis of USP5 and STING expression in WT and Usp5-/-Sting-/- iBMMs. (B) RT-qPCR analysis of Ifnb, Isg54, and Ccl5 in WT and Usp5-/-Sting-/- iBMMs, following infection with SeV (0.1 MOI) for 0, 6, and 12 h. (C) The schematic diagram indicates the principle of in situ PLA measuring endogenous USP5-IRF3 protein interactions in cells. (D) Immunoblot analysis of the expression of IRF3 truncation mutants. (E) Immunoblot analysis of the effect of USP5 on endogenous K48 and K63 ubiquitination of IRF3 following infection with SeV (0.1 MOI) for 8 h. Data are representative of 3 independent experiments (A, B, D, and E). Mean ± SEM, statistical analysis was performed using unpaired two-tailed Student’s t-test (B), *p<0.05, **p<0.01, and ***p<0.001 indicate the significant differences. Created with Biorender.com. (TIF) [file ppat.1012843.s004.tif]
