# Supplementary material for: USP5 inhibits anti-RNA viral innate immunity by deconjugating K48-linked unanchored and K63-linked anchored ubiquitin on IRF3
Source: PLoS Pathog. 2025 Jan 6;21(1):e1012843. doi: 10.1371/journal.ppat.1012843 (PMC11737852; doi:10.1371/journal.ppat.1012843)
Supplement: Attachment — Submitted filename: Response to Reviewers.docx [file ppat.1012843.s007.docx]

**Part I – Answer to reviewer’s summary**

***Reviewer #1:*** *Here the authors note from published work that the deubiquitinase USP5 is downregulated during RNA virus infections. They confirm this finding in several RNA virus infections and examine whether this has functional consequences. They find that USP5 overexpression increases infections while its knockout promotes infection. They observe an inverse correlation between USP5 expression and IFNb induction, suggesting that USP5 negatively regulates interferon induction during infection. They narrow in on IRF3 by using IFNb promoter reporter assays with overexpression of RIG-I/MDA5 signaling pathway components. Direct interaction of USP5 and IRF3 that is increased by infection was confirmed with several independent assays. Mechanistically, they show that USP5 decreases K63- and K48-linked polyubiquitination of IRF3 and that inhibition of IFNb induction and increase of viral infection is dependent on USP5 catalytic activity. The data are clear and convincing in support of the conclusion that USP5 deubiquitinates IRF3 to suppress its IFN-induction activity. This finding is significant because mechanisms limiting IFN induction are important for preventing excessive inflammation and tissue damage. Aside from minor suggestions and one major issue with data interpretation/writing as noted in point 4 below, I support the publication of this manuscript.*

**Response:** Thank you very much for your positive comments and valuable suggestions for our manuscript. We have carefully checked the written contents and revised the bar graphs. The point-by-point responses are listed below. We think that we have addressed all your concerns.

***Reviewer #2:*** *The study by Qiao et al. entitled “USP5 restricts an-RNA viral innate immunity by deconjugating K48-linked unanchored and K63-linked anchored ubiquitin on IRF3” described a novel role of USP5 in regulating IRF3-dependent anti-RNA viral innate immunity. They have not only demonstrated a comprehensive regulatory mechanism that USP5 utilizes its dual-cleavage activity to deubiquitinate IRF3, but also provided a potential target for treating RNA virus infectious diseases. Overall, this is an interesting study with convincing results.*

**Response:** We sincerely thank you for the comments and suggestions. These comments are very helpful in improving our manuscript. We have added more experimental data and carefully revised the manuscript. The point-by-point responses to all the major and minor concerns are provided below.

***Reviewer #3:*** *In this manuscript, the authors reported that the deubiquitinase USP5 is significantly downregulated during the host's innate immune response against RNA viruses, in an IFNAR-dependent manner. USP5 binds to IRF3 and efficiently removes both K63-linked anchored polyubiquitin chains and K48-linked unanchored polyubiquitin chains from IRF3. This action inhibits IRF3-driven transcription of IFN-β and the induction of IFN-stimulated genes (ISGs). The authors further demonstrated that USP5 negatively regulates IRF3-induced antiviral immune responses through its DUB activity. Overall, this is an interesting study with good novelty, which offers valuable insights into the regulation of USP5-mediated immune responses. However, several issues need to be addressed before consideration of publication.*

**Response:** Thank you for your positive comments and valuable suggestions, which indeed have helped us improve the manuscript. Based on your suggestions, we have performed experiments and provided more discussions. The point-by-point responses are shown below. We think that we have addressed all your concerns.

**Part II – Answer to the reviewer’s major points**

**Reviewer #2:**

*1. The authors should add WB results for confirming the overexpression, knockdown, and knockout of USP5 in A549 cells and iBMMs. These results should be displayed adjacently to the corresponding functional experiments or included them in the supplementary figures.*

**Response:** Thank you for your suggestions. We have supplemented the corresponding WB results in the revised Figs. S2D, S2G, and S4A to confirm the knockout or overexpression of USP5 in A549 cells, as well as the double knockout of USP5 and STING in iBMMs.

*2. In Figures S2A and S2B, the GFP fluorescence intensity is weak, and the differences is not so oblivious. It is recommended to extend the infection time or increase the viral infection MOI to enhance GFP intensity.*

**Response:** Thank you for your comments and suggestions. Accordingly, we extended the viral infection time, obtained better results, and replaced the original Figs. S2A and S2B with the improved figures. Although more cells were infected with SeV and VSV, fewer attached cells were observed in brightfield images, which may be associated with the function of USP5 in facilitating apoptosis during inflammation (PMID: 37534934).

**Reviewer #3:**

*1. Can the authors demonstrate which lysine residue of IRF3 undergoes deubiquitination by USP5?*

**Response:** We are deeply grateful for your valuable suggestion. According to the previous studies, IRF3 undergoes K63-linked ubiquitination at lysine 98 and lysine 313 (PMID: 30100102 and 37366613). The K63-linked ubiquitination of IRF3 at lysine 313 serves as a signal to promote CALCOCO2-dependent selective macroautophagy (PMID: 37366613), while K63-linked ubiquitination of IRF3 at lysine 98 activates IRF3 for its transcriptional function (PMID: 30100102). Therefore, lysine 98 of IRF3 is a possible site at which IRF3 undergoes deubiquitination by USP5. K48-linked unanchored polyubiquitination is connected to the substrate through non-covalent interactions (PMID: 24882218), meaning that the lysine residue of the substrate is not a necessary condition for its occurrence (PMID: 20403326). Although previous studies have established the role of unanchored ubiquitination in promoting IFN-I signaling activation (PMID: 38970666), the specific sites in IRF3 where unanchored ubiquitination interacts with its substrate have not yet been reported.

Therefore, specific amino acid residues (e.g., lysines for K63-linked Ub or some hydrophobic residues for K48-linked Ub) undergoing deubiquitination by USP5 need to be further investigated.

*2. Commercial antibodies for K48- and K63-linked polyubiquitination are available. It is good for the authors to detect endogenous IRF3 and its endogenous K48 and K63 ubiquitination using these antibodies?*

**Response:** Thanks for your comments and suggestions. We totally agree that using commercial antibodies to detect endogenous K48 and K63 ubiquitination on IRF3 is necessary. We checked the effect of USP5 on endogenous K48 and K63 ubiquitination of IRF3 during SeV infection and found that overexpression of USP5 significantly reduced both endogenous K48 and K63 ubiquitination on IRF3 (revised Fig. S4E). These results make our conclusion that USP5 inhibits IRF3 ubiquitination more convincing.

**Part III – Answer to the reviewer’s minor points**

**Reviewer #1:**

*1. The use of the word “restricts” in the title could be replaced by “inhibits” or “decreases.” The word restrict is usually used in this field in relation to restriction of virus infections (as opposed to restriction of innate immunity pathways that restrict virus infection as it is used here).*

**Response:** Thank you for your suggestions. We agree that the word 'restricts' in the title was not the best choice. We have changed the title to “USP5 inhibits anti-RNA viral innate immunity by deconjugating K48-linked unanchored and K63-linked anchored ubiquitin on IRF3”.

*2. All bar graphs need to be re-made with symbols/colors that make more sense at first glance. The Key is confusing as open symbols are paired with solid bars and vice versa, making interpretation difficult.*

**Response:** We sincerely thank you for the comments. We have re-made all bar graphs in the revised figures by using more sensible symbols/colors.

*3. The examination of STING KO cells in Fig 3 seems out of place and would not be a logical first place to start in terms of mechanistic examinations. This should be moved to later in the manuscript as a negative control or should be placed in the supplement.*

**Response:** Thank you for your comments and suggestions. We have relocated the data from STING KO cells to the revised Fig. S4B, and the associated results description has also been moved to the beginning of the next paragraph to enhance the narrative logic (lines 191-199).

*4. Lines 180-190 describe the opposite of what the data show, and the conclusion at the end of this paragraph is the opposite of what they state in the previous lines. These are major mistakes in writing that are in opposition to the overall conclusions of the manuscript.*

**Response:** Thanks for your careful reading and valuable suggestions. We sincerely apologize for the oversights in writing mistakes. We have corrected the results description in the revised manuscript (lines 177-188).

*5. Line 192: This may be better written as “To* *explore roles of USP5 in RIG-I signaling, we assessed roles of…”*

**Response:** We sincerely appreciate your suggestion. We have made the revision according to your wonderfully written advice (line 200).

*6. In Figure 5G, aren’t all of the ubiquitin chains shown in these blots expected to be anchored to IRF3 since it’s IP’d? It is thus confusing that the authors conclude that USP5 removes certain unanchored chains. Please clarify/explain further.*

**Response:** We are deeply grateful for your insightful feedback on our manuscript. We sincerely apologize for the oversight in not providing a clear description for Fig. 5G. This figure indeed demonstrates that USP5 removes both K48- and K63-linked polyubiquitination from IRF3. However, it is important to note that this polyubiquitination includes both anchored and unanchored types. The binding between unanchored Ub and its substrate via the non-covalent bonds (PMID: 24882218 and 20403326), which remain intact during IP procedures until the denaturing buffer is used. Therefore, the conventional ubiquitination assays cannot distinguish between anchored and unanchored ubiquitination. In light of this, we have further evaluated the impact of USP5 on both anchored and unanchored polyubiquitination of IRF3 (Figs. 6A and 6B). We adhered to the protocol for isolating endogenous unanchored polyubiquitin chains, as established in previous studies (PMID: 24882218 and 20403326), to separate the unanchored polyubiquitin chains and then added the denaturing agents to further assess the anchored polyubiquitin chains. The protocol for the detection of unanchored ubiquitination has been supplemented in the revised manuscript (lines 445-457) to give readers an easier understanding.

**Reviewer #2:**

*1. The manuscript contains several grammatical errors and types. The authors should check the manuscript carefully by themselves or use professional editing services for assistance.*

**Response:** We appreciate your comments. We have already carefully checked and corrected the grammar errors and typos by using professional editing services for assistance.

*2. In line 33, ‘IFNAR’ is mentioned for the first time, so its full name should be added*.

**Response:** Thanks for your suggestion. We have added the full name of IFNAR when it is mentioned for the first time in the revised manuscript (lines 33-34).

*3. In lines 149-151, to enhance readability, the text could be compressed by not repeating details and moving technical issues to the Materials and methods section or Figure legends.*

**Response:** We sincerely thank you for the comments and suggestions. We have corrected the description in the revised manuscript (lines 151-153).

*4. Please provide the information in the methods section regarding the concentration of MG132.*

**Response:** Thank you for your suggestions. We have added the concentration of MG132 in the methods section in the revised manuscript (lines 441-442).

*5. Please discuss the potential role of unanchored ubiquitin in regulating host antiviral immunity against DNA viruses, since the cGAS-STING signaling pathway also requires IRF3 for induction of type I interferon.*

**Response:** We sincerely thank you for your valuable suggestions. Our results show that Ub(K48)-G76A significantly enhanced IRF3-induced IFN-β and ISRE transcription activities (Fig. 6C). IRF3, as a key transcription factor triggering antiviral immune response in the cGAS-STING signaling pathway, plays an important role in regulating host antiviral immunity against DNA viruses (PMID: 32424334). Consequently, during DNA virus infection, unanchored ubiquitination may promote antiviral immunity by targeting IRF3, although other substrates, including cGAS and STING themselves, are also potentially targeted by the unanchored polyubiquitin.

**Reviewer #3:**

*1. Figure 1F (left): the authors reported that viral infection can downregulate USP5 protein levels. However, in WT group, VSV upregulated Usp5 levels at time point 4 hrs. Please explain. In addition, total STAT1 levels were missing in Figure 1F.*

**Response:** Thanks for your insightful questions. We checked the expression of USP5 at 4 h after VSV infection in other results and found that upregulation of USP5 levels in response to VSV occurred in only one instance. To further verify this, we conducted additional experiments and verified that there was no significant upregulation of USP5 expression following VSV infection. Consequently, we have replaced Fig. 1F (left) with new results to ensure the accuracy and reliability of our findings. In addition, the total STAT1 levels were also added in the revised Fig. 1F.

*2. Figure 2C, 2D: does USP5 overexpression or knockout affect virus titers?*

**Response:** We appreciate your evaluation and valuable comments. We wholeheartedly agree with you that the effect of USP5 overexpression or knockout on virus titer should also be checked. Firstly, we collected the supernatant from the Ctrl and USP5-overexpressing A549 cells post VSV infection for 24 h and measured the supernatant virus titer by using TCID_50_. We found that USP5 overexpression significantly increased the VSV titer (revised Fig. 2D). Next, we performed another TCID_50_ assay to analyze the effect of USP5 knockout on virus titer and discovered that USP5 knockout significantly reduced the VSV titer (revised Fig. 2F).

*3. Figure 3A-3C: doe**s USP5 overexpression or knockout regulate basal expression levels of IFNB and ISGs?*

**Response:** We appreciate your evaluation and valuable comments. In our analysis of the effects of USP5 on the basal expression levels of IFNB and ISGs (data are shown as below), we found that overexpression of USP5 did not significantly alter these basal levels. Nevertheless, we noted slight increasing trends in the basal expression levels of IFNB and ISGs in the *USP5^-/-^* cells, although there was no statistical significance.

Fig 1. The effects of USP5 on the basal expression levels of IFNB and ISGs.

**(A)** RT-qPCR analysis of *IFNB*, *ISG54*, and *CCL5* genes in the Ctrl and Myc-USP5 overexpressed A549 cells.

**(B)** RT-qPCR analysis of *IFNB*, *ISG54*, and *CCL5* in WT and *USP5*^-/-^ A549 cells.

Data are representative of three independent experiments (A and B). Mean ± SEM, statistical analysis was performed using unpaired two-tailed Student’s *t*-test (A and B), *p* < 0.05 indicates the significant differences.

*4. Figure 3E: the p-STAT1 bands in Figure 3E are quite different from those in Figure 3D. Why? I would suggest to repeat Figure 3E to get clearer data.*

**Response:** We sincerely appreciate your questions and comments. The variation in band intensity is attributed to the use of two distinct detection methods—chemiluminescence (ECL, Millipore) and the LI-COR Odyssey CLx imaging system—to assess the p-STAT1 bands across two separate experiments. We sincerely apologize for the oversight in not standardizing the detection methods. To address this issue, we have repeated this experiment using chemiluminescence for the detection of all bands and replaced the original Fig. 3E with new results (revised Fig. 3D).

*5. Figure 4D: it is difficult to observe the staining of both USP5 and IRF3 in Figure 4D.*

**Response:** The principle of *in situ* PLA detection capitalizes on the proximity effect to identify proteins that have interacted with each other. The detailed procedure is schematically represented in the revised Fig. S4C, where the red fluorescence aggregates correspond to USP5-IRF3 interactions. Due to the use of secondary antibodies conjugated with PLA probes in this assay, the individual localization of USP5 and IRF3 cannot be discerned. Additionally, to ensure clear observation of the red foci, we have replaced the mock group with a more representative field in the revised Fig. 4D.

*6. The authors reported that RNA viruses downregulate USP5 expression and that USP5 promotes RNA virus infection. How does USP5 affect DNA viruses?*

**Response:** We appreciate your insightful question. DNA viruses can also trigger the expression of IFN-I via the cGAS-STING signaling pathway (PMID: 32668227). Our results (revised Fig. 1D) have demonstrated that poly(dA:dT) and IFN-I markedly suppress the expression of USP5, suggesting that DNA viruses may also downregulate USP5 expression. IRF3 plays a very important role in the cGAS-STING signaling pathway, which is crucial for the host to defend against DNA virus infections (PMID: 32424334). Consequently, USP5 might inhibit the host's immune response to DNA viruses by targeting IRF3. However, as an important deubiquitinating enzyme, USP5 has multiple substrates (PMID: 31756387). Therefore, it requires further investigation to determine whether there are other substrates of USP5 involved in the regulation of the host anti-DNA viral immune response.

*7. In Figure 3F, the number of cells in each group varies considerably. Does USP5 regulate cell proliferation?*

**Response:** We appreciate your evaluation and valuable comments on our manuscript. We evaluated the effect of USP5 overexpression on A549 cell proliferation using the CCK8 assay. No significant differences in growth were observed between control cells and the cells overexpressing USP5 over a 72-hour period (data are shown as below), indicating that the role of USP5 in modulating host cell resistance to viral infection is not mediated through the regulation of cell proliferation.

Fig 2. USP5 has no influence on cell proliferation.

The CCK8 assay analyzes the effect of USP5 overexpression on A549 cell proliferation. Data are representative of at least 3 independent experiments. Mean ± SEM, statistical analysis was performed using unpaired two-tailed Student’s *t*-test, ns indicates not significant.

8. *The description of results from lines 183 to 188 contains inaccuracies.*

**Response:** Thank you very much for your efforts and comments on our manuscript. We sincerely apologize for the oversight in the results description. We have corrected the results description in the revised manuscript (lines 180-186).
